# Determinants of improvement of left ventricular mechano-energetic efficiency in hypertensive patients

**DOI:** 10.3389/fcvm.2022.977657

**Published:** 2022-07-28

**Authors:** Maria Lembo, Valentina Trimarco, Maria Virginia Manzi, Costantino Mancusi, Giovanni Esposito, Salvatore Esposito, Carmine Morisco, Raffaele Izzo, Bruno Trimarco

**Affiliations:** ^1^Department of Advanced Biomedical Sciences, Federico II University of Naples, Naples, Italy; ^2^Department of Neurosciences, Federico II University of Naples, Naples, Italy

**Keywords:** arterial hypertension, beta-blockers, metabolic control, myocardial efficiency, left ventricular function

## Abstract

**Background:**

Arterial hypertension, especially when coexisting with other cardiovascular risk factors, could determine an imbalance between myocardial energetic demand and altered efficiency, leading to an early left ventricular (LV) systolic dysfunction, even in terms of echo-derived mechano-energetic efficiency indexed for myocardial mass (MEEi). We aim to analyse an improvement in LV MEEi, if any, in a population of hypertensive patients with a long-term follow-up and to identify clinical, metabolic and therapeutic determinants of LV MEEi amelioration.

**Materials and methods:**

In total, 7,052 hypertensive patients, followed-up for 5.3 ± 4.5 years, enrolled in the Campania Salute Network, underwent echocardiographic and clinical evaluation. LV MEEi was obtained as the ratio between stroke volume and heart rate and normalized per grams of LV mass and ΔMEEi was calculated as difference between follow-up and baseline MEEi. Patients in the highest ΔMEEi quartile (≥0.0454 mL/s/g) (group 1) were compared to the merged first, second and third quartiles (<0.0454 mL/s/g) (group 2). METS-IR (Metabolic Score for Insulin Resistance), an established index of insulin sensitivity, was also derived.

**Results:**

Patients with MEEi improvement experienced a lower rate of major cardiovascular events (*p* = 0.02). After excluding patients experiencing cardiovascular events, patients in group 1 were younger (*p* < 0.0001), less often diabetic (*p* = 0.001) and obese (*p* = 0.035). Group 1 experienced more frequently LV mass index reduction, lower occurrence of LV ejection fraction reduction, and had a better metabolic control in terms of mean METS-IR during the follow-up (all *p* < 0.0001). Beta-blockers were more often used in group 1 (*p* < 0.0001) than group 2. A logistic regression analysis showed that younger age, lower mean METS-IR values, more frequent LV mass index reduction and therapy with beta-blockers were significantly associated with LV MEEi improvement, independently of presence of diabetes and obesity.

**Conclusion:**

Metabolic control and therapy with beta-blockers could act in a synergic way, determining an improvement in LV MEEi in hypertensive patients over time, possibly confining cardiac damage and hampering progression toward heart failure.

## Introduction

Arterial hypertension, in particular when associated with additional cardiovascular risk factors, induces an imbalance in cardiac metabolism by increasing myocardial energetic demand and impairing energetic efficiency. This condition represents an early cardiac damage, which increases the risk of progression toward heart failure (1–4).

Ultrasound assessment represents an accurate and wide available methodology to identify this early left ventricular (LV) functional impairment in the hypertensive setting (5). Indeed, echo-derived mechano-energetic efficiency indexed for myocardial mass (MEEi), obtained as the ratio between stroke work and oxygen consumption indexed for LV mass, is an easily applicable and sensitive parameter for the evaluation of LV performance. MEEi was demonstrated to be a strong predictor of cardiovascular events and heart failure onset in multiple settings, including arterial hypertension (6–9). In fact, low MEEi values were recently demonstrated to be able to identify patients more prone to develop LV systolic dysfunction, in terms of LV ejection fraction (LVEF) reduction, in a group of hypertensive patients with optimal blood pressure (BP) control during a long-term follow up (10).

On the other hand, it is still unclear whether the control of risk factors by modification of life habits and diet and effective therapeutic strategies can improve an impaired MEEi in hypertensive patients. Indeed, it has been demonstrated that both effective antihypertensive therapy and weight loss could induce a positive remodeling of the left ventricle over time, with a regression of LV mass and amelioration of LV systolic function (11–13). In addition, evidence showed that reduction of body weight and insulin resistance were associated with a reverse of LV morphological and functional abnormalities (14). Metabolic Score for Insulin Resistance (METS-IR) is an established index of insulin resistance and metabolic control (15), but an association between this index and LV performance in terms of MEEi was never reported.

Thus, we assessed the time-course of MEEi in a population of hypertensive patients with a long-term follow up, to test the possibility to improve an impaired LV MEEi and to identify clinical, metabolic, even in terms of METS-IR, and pharmacological determinants of such an increase in MEEi, if any, and its relevance on cardiovascular prognosis.

## Materials and methods

### Study population

Hypertensive patients, enrolled in the Campania Salute Network, were evaluated during a long-term follow-up. The Campania Salute Network was previously described in detail and was approved by the Federico II University Hospital Ethic Committee (ClinicalTrials.gov Identifier: NCT02211365) (16). All patients gave signed written informed consent (17).

Inclusion criteria were: (1) age more than 18 years; (2) available follow-up with echo assessment ≥ 12 months; (3) no history of prevalent cardiovascular disease, including myocardial infarction, angina pectoris, coronary or carotid revascularization procedures, stroke, transitory ischemic attack, congestive heart failure, clinically relevant heart valvular disease (more than mild valve regurgitations and any stenosis), chronic kidney disease more than stage 3; (4)available baseline echocardiographic and carotid ultrasound assessment; 4) LVEF > 50% at baseline echocardiographic exam; (5) ability to give informed consent.

The rate of major cardiovascular events, that happened during the follow-up period, was related to the first occurrence. Major cardiovascular events developed during the follow-up included myocardial infarction, coronary or carotid revascularization procedures, stroke and transitory ischemic attack.

Diagnosis of arterial hypertension was established on the basis of current ESC/ESH guidelines (18). Heart rate, systolic, and diastolic BP were collected in sitting position, after 5 mins of rest, using a semiautomatic oscillometric sphygmomanometer with cuffs of appropriate size. Measurements were repeated in supine position after the echocardiographic exam. Optimal office BP control was defined according to ESC/ESH guidelines for management of arterial hypertension (18). Pulse pressure was measured as the difference between systolic BP and diastolic BP (19).

Glomerular filtration rate was measured with the chronic kidney disease epidemiology collaboration (CKD-EPI) equation (20).

Diabetes was established for values of fasting plasma glucose > 126 mg/dl or specific antidiabetic treatment (21). Obesity was identified for values of body mass index ≥ 30 kg/m^2^. Weight loss was described as a reduction in body weight ≥ 5% at the final available visit, compared to initial value, as previously reported (11).

Metabolic evaluation was assessed by an insulin resistance surrogate, METS-IR, computed as follows: METS-IR = {ln [2 × Fasting plasma glucose (mg/dL) + Triglycerides (mg/dL)] × Body Mass Index (kg/m^2^)/ln [HDL-Cholesterol (mg/dL)]} (15).

### Ultrasound assessment

All echocardiographic exams were performed at the Hypertension Outpatient Clinic of the Federico II University in Naples, using a standardized protocol. All measurements were evaluated according to the latest consolidated convention (22, 23).

Left ventricular mass was estimated from a necropsy-validated formula and normalized for height in meters to the power of 2.7 (LV mass index) (24, 25). LV hypertrophy was defined for values of LV mass index > 47 g/m^2^.^7^ in women and >50 g/m^2^.^7^ in men (26, 27). LV mass index reduction was defined for a decrease of at least of 5 g/m^2^.^7^ from baseline at the end of the follow up, as previously reported (12).

Left ventricular ejection fraction and stroke volume were computed as the difference between LV end- diastolic and end-systolic volume by the z-derived method and indexed for height to the power of 2.04 (28, 29). LVEF was considered reduced for values less than 50% at final echo or for a LVEF reduction of at least 10 percentage points compared to baseline (10, 30, 31).

Left ventricular myocardial mechano-energetic efficiency was computed as the ratio between stroke volume and heart rate and normalized per grams of LV mass, and expressed in mL/s/g (MEEi), as previously reported (32). Delta MEEi was calculated as the difference between follow-up and baseline MEEi values.

Left atrial volume was estimated according to previously validated formula and indexed for height powered to 2 (33).

Carotid ultrasound was assessed in supine position. The intima–media thickness was measured as the distance between lumen-intima and media-adventitia interface in up to 2 arterial walls, on both near and far walls of distal common carotid (1 cm), bifurcation and proximal internal carotid artery of both sides and carotid plaques were defined for intima–media thickness values > 1.5 mm (34).

### Statistical analysis

Categorical variables were expressed as number (percentage) and continuous variables as mean ± standard deviation or median [interquartile range] when not normally distributed. Chi-square test and Student’s *t*-test were used to test differences in dichotomous/categorical and continuous covariates, respectively.

Delta MEEi quartiles were computed: first quartile for delta MEEi ≤ −0.0354 mL/s/g, second quartile ranging from −0.0354 to ≤0.0028 mL/s/g, third quartile from 0.0028 to <0.0454 mL/s/g, fourth (highest) quartile ≥ 0.0454 mL/s/g. The study population was divided into two groups: patients who presented an improvement in MEEi as highest delta MEEI quartile (group 1), were compared to patients of the merged first, second and third delta MEEi quartiles (group 2). Univariate logistic regression analyses were assessed to verify important variables for delta MEEi improvement.

A logistic regression model was built including the significant variables derived from univariate correlations in order to identify possible factors associated with LV MEEi improvement in terms of highest delta MEEi quartile. Calculation of tolerance and variance inflation was performed by linear modeling and collinearity was considered acceptable for variance inflation factor less than 3.

In all analyses a *p*-value < 0.05 was considered statistically significant. All statistical analyses were performed using SPSS Statistics 26 software (IBM Corp, Armonk, NY, United States).

## Results

Patients were followed-up for 5.3 ± 4.5 years and during this period major cardiovascular events occurred in 165 patients with a prevalence of 1.5% among those showing an improvement of MEEi which was significantly lower when compared with the one of patients who did not experience any increase or even a decrease in MEEi (2.5%, *p* = 0.02).

**FIGURE 1:**
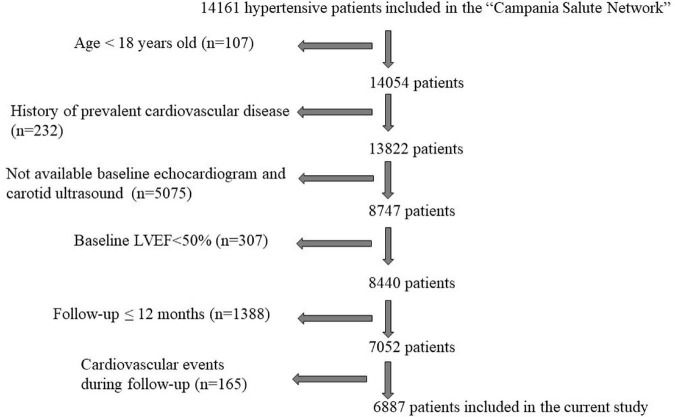
Flow chart describing the selection of hypertensive patients from the “Campania Salute Network” for the present study. LVEF, left ventricular ejection fraction.

After excluding patients experiencing major cardiovascular events during the follow-up, the study population included 6,887 hypertensive patients (Figure 1), 58% males, 10% affected by diabetes mellitus and 26% obese.

The baseline characteristics of the two study groups, divided according to the values of delta MEEi as reported in the “Materials and methods” section, are summarized in Table 1. Patients in the highest delta MEEi quartile (group 1) were younger, less often diabetic and obese, had higher baseline values of systolic and diastolic BP and heart rate than patients of the merged first, second and third delta MEEi quartiles (group 2). At baseline, METS-IR was significantly lower in patients of group 1 compared to group 2. Those patients had also baseline higher echocardiographic-derived relative wall thickness, reduced stroke volume and LV EF, and lower values of left atrial volume index.

**TABLE 1:** Baseline characteristics of the study population.

	Merged first, second and third delta MEEI quartiles *n* = 5,164	Highest delta MEEI quartile improvement *n* = 1,723	*P*-value
Age (year)	53.8 ± 11.2	52.5 ± 10.8	<0.0001
Male sex (%)	2,977 (57.6)	963 (55.9)	0.206
Systolic BP (mmHg)	142.0 ± 17.9	144.2 ± 18.9	<0.0001
Diastolic BP (mmHg)	88.4 ± 10.9	90.6 ± 11.0	<0.0001
Pulse pressure (mmHg)	53.6 ± 14.6	53.6 ± 14.9	0.906
Heart rate (bpm)	72.1 ± 10.4	81.4 ± 11.7	<0.0001
Body mass index (kg/m^2^)	27.8 ± 4.3	27.6 ± 4.1	0.022
Smoke habits (%)	1,072 (20.8)	340 (19.7)	0.370
Diabetes (%)	535 (10.4)	132 (7.7)	0.001
Obesity (%)	1,350 (26.1)	406 (23.6)	0.035
Fasting plasma glucose (mg/dl)	98.6 ± 22.9	97.1 ± 20.8	0.011
Serum total cholesterol (mg/dl)	206.0 ± 38.9	206.2 ± 39.1	0.856
Serum HDL Cholesterol (mg/dl)	50.4 ± 12.9	51.1 ± 12.9	0.041
Serum triglycerides (mg/dl)	136.2 ± 75.4	132.4 ± 75.5	0.071
Serum uric acid (mg/dl)	5.2 ± 1.5	5.1 ± 1.5	0.152
METS-IR	41.7 ± 8.0	41.0 ± 7.6	0.001
CKD-EPI	77.9 ± 15.8	78.2 ± 15.4	0.015
LV hypertrophy (%)	1870 (36.2)	669 (38.9)	0.051
LV mass index (g/m^2^.^7^)	48.0 ± 9.1	46.5 ± 8.4	0.273
Relative wall thickness	0.38 ± 0.04	0.39 ± 0.04	<0.0001
Left atrial volume index (ml/m^2^)	13.1 ± 2.6	12.8 ± 2.4	<0.0001
Stroke volume index (ml/m^2^)	39.8 ± 4.9	39.1 ± 5.0	<0.0001
LVEF%	66.4 ± 3.7	65.9 ± 3.8	<0.0001
MEEi (mL/s/g)	0.35 ± 0.07	0.31 ± 0.06	<0.0001
Intima-media thickness (mm)	1.62 ± 0.73	1.54 ± 0.68	<0.0001

BP, blood pressure; CKD-EPI, chronic kidney disease epidemiology collaboration; LV, left ventricular; LV EF, left ventricular ejection fraction; MEEi, mechano-energetic efficiency indexed for myocardial mass.

Despite patients of group 1 and group 2 reached comparable BP values during the follow-up, patients in group 1 experienced a larger reduction in BP values since they had higher BP values at baseline; indeed, patients of group 1 presented a higher delta reduction in both systolic BP and diastolic BP from baseline to the end of the follow-up than group 2, [median (interquartile range)] [delta systolic BP = −8 (−22 to 5) mmHg vs. −5 (−20 to 8) mmHg, *p* < 0.0001; delta diastolic BP = −10 (−18 to 0) mmHg vs. −5 (−15 to 2) mmHg, *p* < 0.0001]. In addition, patients in group 1 had a higher prevalence of LV mass index reduction and a better metabolic control in terms of METS-IR, lower mean serum triglycerides, mean fasting glucose plasma levels and higher mean HDL cholesterol levels, while lower BMI (body mass index) values during the follow-up (Table 2). Furthermore, at the end of the follow-up they showed higher values of LVEF and stroke volume index in comparison to group 2 on account of a statistically significant lower rate in the occurrence of a reduction in LVEF, as defined in the “Materials and methods” section, in comparison to patients of group 2 (0.3 vs 1.6%, *p* < 0.0001).

**TABLE 2:** Follow-up data and treatments.

	Merged first, second and third delta MEEI quartiles	Highest delta MEEI quartile improvement	*P*-value
	*n* = 5,164	*n* = 1,723	
Mean systolic BP during follow up (mmHg)	137.2 ± 12.7	136.9 ± 12.3	0.387
Mean diastolic BP during follow up (mmHg)	84.3 ± 7.4	84.4 ± 7.0	0.432
Mean pulse pressure during follow up (mmHg)	53.5 ± 12.5	53.6 ± 12.6	0.75
Mean heart rate during follow up (bpm)	75.3 ± 8.0	73.0 ± 7.6	0.199
Mean body mass index during follow up (kg/m^2^)	27.8 ± 5.1	27.6 ± 4.9	0.027
Weight loss (%)	810 (15.7)	301 (17.5)	0.082
Mean weight during follow up (kg)	78.4 ± 14.2	77.3 ± 13.3	0.003
Mean fasting plasma glucose during follow up (mg/dl)	100.4 ± 21.7	98.8 ± 17.7	0.005
Mean serum total cholesterol during follow up (mg/dl)	200.4 ± 32.9	200.5 ± 32.5	0.859
Mean serum HDL cholesterol during follow up (mg/dl)	50.8 ± 11.5	51.9 ± 11.5	0.001
Mean serum triglycerides during follow up (mg/dl)	133.2 ± 62.2	128.4 ± 57.8	0.005
Mean serum uric acid during follow up (mg/dl)	5.2 ± 1.3	5.2 ± 1.2	0.833
Mean METS-IR during follow up	41.6 ± 9.0	40.7 ± 8.6	< *0*.0001
Mean CKD-EPI during follow up	79.3 ± 14.2	80.0 ± 13.7	0.078
LV mass index at the end of follow up (g/m^2.7^)	48.0 ± 9.1	47.0 ± 9.0	< *0*.0001
LV mass index reduction at the end of follow up (%)	261 (5.1)	218 (12.7)	< *0*.0001
Relative wall thickness at the end of follow up	0.39 ± 0.04	0.38 ± 0.03	< *0*.0001
Left atrial volume index at the end of follow up (ml/m^2^)	14.1 ± 2.9	13.9 ± 2.6	0.107
Stroke volume index at the end of follow up (ml/m^2^)	39.6 ± 4.9	40.8 ± 4.7	< *0*.0001
LVEF at the end of follow up (%)	66.0 ± 3.8	67.3 ± 3.6	< *0*.0001
LVEF reduction (%)	83 (1.6)	6 (0.3)	< *0*.0001
MEEI at the end of follow up (mL/s/g)	0.32 ± 0.06	0.38 ± 0.06	< *0*.0001
Intima-media thickness at the end of the follow up (mm)	1.78 ± 0.78	1.71 ± 0.74	0.001
BP control at the end of the follow up (%)	2,952 (57.2)	1,015 (58.9)	0.216
Medication at least 50% of control visits	1.64 ± 1.03	1.64 ± 1.04	0.921
Beta blockers	1,246 (24.1)	561 (32.6)	< *0*.0001
Anti-renin-angiotensin-aldosterone system	4,221 (81.7)	1,399 (81.2)	0.615
Diuretics	2,229 (43.2)	737 (42.8)	0.779
dihydropyridine Calcium channel blockers	1,383 (26.8)	395 (22.9)	0.001
Statins	929 (18.3)	325 (19.2)	0.517
Antiplatelet therapy	894 (17.6)	261 (15.3)	0.028

BP, blood pressure; CKD-EPI, chronic kidney disease epidemiology collaboration; LV, left ventricular; LV EF, left ventricular ejection fraction; MEEi, mechano-energetic efficiency indexed for myocardial mass.

Regarding treatment, beta blockers were more often used in group 1, whereas antiplatelet therapy and dihydropyridine Calcium channel blockers were more often used in group 2.

Univariate logistic regressions showed that age, diabetes mellitus, obesity, mean fasting plasma glucose, mean triglycerides, mean HDL cholesterol levels during the follow up, mean METS-IR, LV mass index reduction, therapy with dihydropyridine Calcium channel blockers, antiplatelet therapy and therapy with beta-blockers present in more than 50% of the follow-up were significantly related to delta LV MEEi improvement (Table 3).

**TABLE 3:** Univariate regression analyses.

	LV MEEi improvement	
	Regression coefficient	*P*-value
Age	0.99	<0.0001
Female sex	1.07	0.202
Mean systolic BP during Follow-up (mmHg)	0.99	0.378
Mean diastolic BP during Follow-up (mmHg)	1.00	0.439
Mean pulse pressure (mmHg)	1.00	0.949
Mean heart rate during follow-up (bpm)	0.99	0.793
Smoke habits (%)	0.94	0.361
Obesity (%)	0.87	0.034
Diabetes (%)	0.72	0.001
Mean body mass index during follow up (kg/m^2^)	0.99	0.024
Mean weight during follow up (kg)	0.99	0.003
Weight loss (%)	1.13	0.081
Mean Fasting plasma glucose during follow up (mg/dl)	1.00	0.005
Mean serum uric acid during follow up (mg/dl)	1.00	0.833
Mean serum Triglycerides during follow up (mg/dl)	1.00	0.006
Mean serum total cholesterol during follow up (mg/dl)	1.00	0.859
Mean serum HDL cholesterol during follow up (mg/dl)	1.01	0.001
Mean METS-IR	0.99	<0.0001
Mean CKD-EPI during follow up	1.00	0.078
LV mass index reduction at the end of follow up (g/m^2^.^7^)	2.72	<0.0001
Number of medications in at least 50% of control visits	1.00	0.921
Anti-renin-angiotensin-aldosterone system	0.97	0.614
Antiplatelet therapy	0.84	0.027
Dihydropyridine calcium channel blockers	0.81	0.002
Beta-blockers	1.52	<0.0001
Statins	1.05	0.504
Diuretics	0.98	0.777

BP, blood pressure; CKD-EPI, chronic kidney disease epidemiology collaboration; LV, left ventricular; LV EF, left ventricular ejection fraction; MEEi, mechano-energetic efficiency indexed for myocardial mass.

**FIGURE 2:**
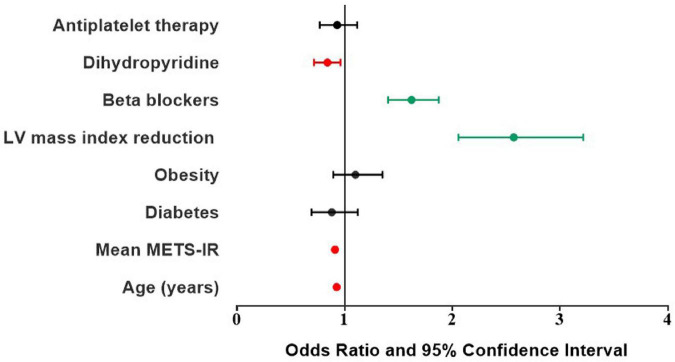
Odds ratio and 95% confidence interval of clinical and therapeutic determinants of delta MEEi improvement. MEEi, indexed mechano-energetic efficiency.

A logistic regression analysis (Table 4) showed that younger age, better metabolic control in terms of METS-IR, more frequent LV mass index reduction and therapy with beta blockers were significantly associated with LV MEEi improvement, independently of the presence of diabetes and obesity, whereas therapy with dihydropyridine Calcium channel blockers was negatively associated with MEEi improvement (Figure 2).

**TABLE 4:** Logistic regression analysis performed for describing determinants of delta MEEi improvement.

	OR	CI	*P*-value
Age	0.99	0.985–0.998	0.010
Mean METS-IR	0.98	0.970–0.994	0.004
Diabetes	0.88	0.692–1.122	0.304
Obesity	1.10	0.894–1.351	0.368
LV mass index reduction	2.57	2.056–3.216	<0.0001
Beta blockers	1.62	1.403–1.874	<0.0001
Dihydropyridine Calcium channel blockers	0.84	0.716–0.982	0.029
Antiplatelet therapy	0.93	0.769–1.116	0.422

BP, blood pressure; CKD-EPI, chronic kidney disease epidemiology collaboration; LV, left ventricular; LV EF, left ventricular ejection fraction; MEEi, mechano-energetic efficiency indexed for myocardial mass.

## Discussion

The current study demonstrated that: (1) an improvement in MEEi in hypertensive patients is achievable; (2) during the follow up the largest MEEi improvement was associated with a significantly lower prevalence of major cardiovascular events; (3) after excluding patients who experienced major cardiovascular events, those with the largest improvement in MEEi were the ones who had the lower rate of reduction in LVEF during the follow-up, (4) better metabolic control, in terms of METS-IR, and higher rate of LV mass reduction were directly associated to LV MEEi improvement during the follow-up, independently of the presence of diabetes and obesity, (5) among treatments, therapy with beta-blockers was significantly and independently associated with LV MEEi improvement.

The possibility to identify the determinants of an amelioration in LV mechanic-efficiency could be of relevant interest, since the recognition of the factors and therapies contributing to preserve LV performance could avoid the progression of cardiac impairment demonstrated to be associated with arterial hypertension, especially when in combination with additional cardiovascular risk factors (2, 35). The combined noxious impact of these cardiovascular risk factors could determine endothelial dysfunction, LV remodeling and hypertrophy, and could alter cardiac metabolism (4, 36–38). This condition could result in an impaired myocardial efficacy of energy utilization and LV MEEi reduction. LV MEEi, a sensitive and easily echo-derived parameter, computed as the ratio between stroke work and oxygen consumption indexed for LV mass, is in fact expression of alterations, possibly present at the same time, involving LV function, morphology and metabolism (32). The latter is related to increased oxygen consumption possibly linked to augmented heart rate, increased LV mass and insulin resistance leading to enhanced fatty acid oxidation (39–41).

Low MEEi values, indeed, were showed to be a reliable predictor of cardiovascular events and heart failure and values below 0.29 of this index were demonstrated to be associated with systolic dysfunction and LVEF reduction (10).

On this basis, in the present study we investigated the possibility of an improvement of LV MEEi in terms of delta MEEi from baseline to the end of a long-term follow-up in a population of hypertensive patients, its clinical relevance and its determinants.

We confirmed that an improvement in MEEi is possible and that it is clinically relevant since patients in the highest delta MEEi quartile experienced a lower rate of cardiovascular events during the follow-up.

Furthermore, in order to exclude the confounding factor related to a reduction of MEEi in the occurrence of major cardiovascular events during the follow-up, we excluded those patients from subsequent analysis.

Left ventricular MEEi improvement of the highest delta quartile (≥0.0454 mL/s/g) means that patients possibly jumped from the lowest quartile of MEEi at baseline to the third or to the highest quartile at the end of the follow-up. In the present study, we observed that patients who experienced an improvement of LV MEEi had at baseline higher BP and heart rate values, they were younger, less often diabetic and obese and had better lipidic and glucose serum profile together with better renal function. At baseline they seem to have a slightly but significant impairment in both LV morphology (higher relative wall thickness) and function (lower LV EF and stroke volume). Mean blood pressure and heart rate values were comparable during the follow-up, meaning that patients in the highest delta MEEi quartile achieved a more pronounced decrease in BP values and heart rate, since they had significantly higher values at baseline. In addition, during the follow-up they maintained a better metabolic control with lower values of serum fasting plasma glucose, triglycerides, lower body weight and BMI, and higher serum HDL-cholesterol. This also resulted in lower values of METS-IR. The latter was demonstrated to be an established index for the evaluation of insulin sensitivity and of cardiovascular risk, containing in its computation information about fasting plasma glucose, serum triglycerides, HDL-cholesterol levels and BMI (42). Furthermore, the improved metabolic control also had an important impact on LV morphology and function. Indeed, patients who experienced an improvement in MEEi had also a higher prevalence of LV mass index reduction and an improvement in both LV EF and stroke volume. The incidence of LVEF reduction, defined as a drop more than 50% at final echo or of at least 10 percentage points compared to baseline values, was significantly lower in patients of group 1 than group 2, thus suggesting that patients who presented an improvement in delta MEEi developed a reduced incidence and progression toward heart failure during the follow-up.

A logistic regression analysis provided additional information. After adjusting for age, mean METS-IR, prevalence of diabetes and obesity, LV mass index reduction, use of beta-blockers, dihydropyridine Calcium channel blockers and antiplatelet therapy, the improvement in MEEi from baseline to the end of the follow-up was significantly associated with younger age, better metabolic control in terms of METS-IR, more frequent LV mass index reduction and therapy with beta blockers, independently of the presence of diabetes and obesity, whereas therapy with dihydropyridine Calcium channel blockers was negatively associated with MEEi improvement.

These findings corroborate previous observations demonstrating the beneficial impact of the reduction of both body weight and BMI and insulin resistance on LV morphology and function and extend them in a population of hypertensive patients with several additional cardiovascular risk factors and a long-term follow-up (11, 12). Indeed, in this setting the metabolic control could achieve an amelioration in LV systolic performance, measured by LV MEEi, early impaired even when LVEF still ranges within normal values. Furthermore, we demonstrated a significant correlation between METS-IR and LV MEEi; the association between METS-IR and improved MEEi remained significant, independently of the presence of obesity and diabetes, thus demonstrating the importance of the impact of insulin sensitivity on myocardial dynamics and morphology even at early stages of metabolic alterations.

Among therapeutic approaches only beta-blockers resulted to have a beneficial effect on LV MEEi improvement. Current guidelines for the management of arterial hypertension suggests the use of combination therapies based on renin-angiotensin-aldosterone system inhibitors, diuretics and dihydropyridine calcium-channel blockers for most of hypertensive patients (18). Nonetheless, the results of the present study suggest that a wider use of beta-blockers could improve LV efficiency and reduce the incidence of cardiovascular complications. In addition, previous evidence indicated that beta-blockers, especially when used in combination with diuretics, could impact negatively on metabolic state, favoring diabetes onset in predisposed individuals (43, 44). The present study suggests, instead, that this class of drugs could have, in a real-life registry, a beneficial effect on cardiac metabolism, even in patients presenting an initial metabolic imbalance. In fact, beta-blockers may act both reducing heart rate and decreasing oxidative metabolism, thus leading to an improvement in cardiac efficiency (45).

Furthermore, it is possible to speculate that the negative association of dihydropyridine calcium channel blockers with the highest delta LV MEEi quartile may be related to the increase in sympathetic discharge that these drugs induce through a baroreceptor reflex, which leads to an increase in heart rate, negatively impacting on MEEi (46).

Thus, both metabolic control and beta-blockers, acting in a synergic way, had a favorable effect on LV function and morphology inducing an improvement in cardiovascular prognosis detectable by the increase in LV MEEi.

## Limitations

Additional sensitive parameters for the evaluation of LV systolic function, such as global longitudinal strain and strain derived myocardial work components, are missing in the present study. Nevertheless, MEEi is a sensitive and well-established parameter of LV systolic performance and has the advantage of being derived from standard echocardiographic assessment without the need of additional software. Moreover, information about which type of antidiabetic therapy was administered in the study population is missing, together with info about serum glycated hemoglobin values. Nonetheless, in the present study we demonstrated that metabolic control in terms of METS-IR was a significant determinant of LV MEEi improvement independently of the presence of diabetes. Furthermore, the Campania Salute Network is an observational registry, and it could possibly be influenced by selection bias. However, all patients underwent the same visits and echocardiographic evaluations and followed the same standardized protocol.

## Conclusion

In a population of hypertensive patients with several cardiovascular risk factors, metabolic control and therapy with beta-blockers were significantly associated with an improvement in LV systolic performance in terms of LV MEEi during a long-term follow-up, thus corroborating the usefulness of this parameter, easily obtainable from standard echocardiographic assessment. LV MEEi improvement was independent of the presence of diabetes and obesity and clinically relevant, as demonstrated by a lower prevalence of major cardiovascular events and occurrence of LVEF reduction. All together our results demonstrate the importance of early obtaining and maintaining adequate metabolic profile and insulin sensitivity in hypertensive patients in order to possibly limit myocardial damage and progression toward heart failure.

## Data availability statement

The raw data supporting the conclusions of this article will be made available by the authors, without undue reservation.

## Ethics statement

The studies involving human participants were reviewed and approved by Federico II University Hospital Ethical Committee. The patients/participants provided their written informed consent to participate in this study.

## Author contributions

ML wrote the manuscript. VT helped in writing the manuscript and data collection. MM and CMa collect the clinical and echo data and performed the statistical analysis. GE, SE, and CMo critically revised the manuscript. RI and BT conceived the idea and critically revised the manuscript. All authors give final approval to the submission of the manuscript.

## Abbreviations

BMI: body mass index
BP: blood pressure
CKD-EPI: chronic kidney disease epidemiology collaboration
LV: left ventricular
LVEF: left ventricular ejection fraction
MEEi: mechano-energetic efficiency indexed for myocardial mass
METS-IR: Metabolic Score for insulin resistance.
